# Spermidine Promotes Human Hair Growth and Is a Novel Modulator of Human Epithelial Stem Cell Functions

**DOI:** 10.1371/journal.pone.0022564

**Published:** 2011-07-27

**Authors:** Yuval Ramot, Stephan Tiede, Tamás Bíró, Mohd Hilmi Abu Bakar, Koji Sugawara, Michael P. Philpott, Wesley Harrison, Marko Pietilä, Ralf Paus

**Affiliations:** 1 Department of Dermatology, Hadassah - Hebrew University Medical Center, Jerusalem, Israel; 2 Department of Dermatology, University of Lübeck, Lübeck, Germany; 3 Department of Physiology, Research Centre for Molecular Medicine, University of Debrecen, Debrecen, Hungary; 4 Universiti Sains Malaysia, Kelantan, Malaysia; 5 Centre for Cutaneous Research, Blizard Institute of Cell and Molecular Science, Barts and the London School of Medicine and Dentistry, Queen Mary University of London, London, United Kingdom; 6 Department of Biotechnology and Molecular Medicine, A. I. Virtanen Institute for Molecular Sciences, Biocenter Kuopio, University of Eastern Finland, Kuopio, Finland; 7 Epithelial Sciences, School of Translational Medicine, University of Manchester, Manchester, United Kingdom; Ludwig-Maximilian-University, Germany

## Abstract

**Background:**

Rapidly regenerating tissues need sufficient polyamine synthesis. Since the hair follicle (HF) is a highly proliferative mini-organ, polyamines may also be important for normal hair growth. However, the role of polyamines in human HF biology and their effect on HF epithelial stem cells *in situ* remains largely unknown.

**Methods and Findings:**

We have studied the effects of the prototypic polyamine, spermidine (0.1–1 µM), on human scalp HFs and human HF epithelial stem cells in serum-free organ culture. Under these conditions, spermidine promoted hair shaft elongation and prolonged hair growth (anagen). Spermidine also upregulated expression of the epithelial stem cell-associated keratins K15 and K19, and dose-dependently modulated *K15* promoter activity *in situ* and the colony forming efficiency, proliferation and K15 expression of isolated human K15-GFP+ cells *in vitro*. Inhibiting the rate-limiting enzyme of polyamine synthesis, ornithine decarboyxlase (ODC), downregulated intrafollicular K15 expression. In primary human epidermal keratinocytes, spermidine slightly promoted entry into the S/G2-M phases of the cell cycle. By microarray analysis of human HF mRNA extracts, spermidine upregulated several key target genes implicated e.g. in the control of cell adherence and migration (*POP3*), or endoplasmic reticulum and mitochondrial functions (*SYVN1*, *NACA* and *SLC25A3*). Excess spermidine may restrict further intrafollicular polyamine synthesis by inhibiting ODC gene and protein expression in the HF's companion layer *in situ*.

**Conclusions:**

These physiologically and clinically relevant data provide the first direct evidence that spermidine is a potent stimulator of human hair growth and a previously unknown modulator of human epithelial stem cell biology.

## Introduction

Polyamines (spermidine, putrescine and spermine) are multifunctional polycationic aliphatic amines, which serve crucial roles in cell survival. Besides serving as nutrients and metabolic regulators [1], [2], polyamines have been implicated as mediators of key cell functions, such as proliferation, migration and differentiation [3]–[7]. Polyamines also stabilize DNA/RNA and modulate DNA replication/transcription [1], [2], [8]–[10], and stabilize membrane and cytoskeletal proteins [11], [12]. Recently a key polyamine, spermidine, has even been hailed as a new longevity agent due to its impact on chromatin-mediated regulation of gene expression [13]. While polyamines are indispensable for cell proliferation, and are needed for the growth of rapidly regenerating tissues and tumors [14], [15], the full spectrum of functions of spermidine in normal *human* tissue physiology remains poorly understood [16].

The hair follicle (HF) is one of the most highly proliferative organs in mammalian biology [17], [18]. Therefore, polyamines have long been suspected to be important for hair growth [19]. For example, inhibiting polyamine synthesis significantly modulates murine hair growth [20], and polyamines have an essential role in determining sheep HF growth and diameter [21]. In addition, a recent study has shown that a topical administration of α-methylspermidine, a stable analogue of spermidine, induced hair growth in telogen phase mice [22]. Surprisingly, however, studies utilizing several transgenic mice lines with altered polyamine metabolism [6], [19], [20], [23]–[28] showed that the most prominent phenotype of these mice was hair loss due to disturbed proliferation of follicular keratinocytes.

In human skin, topical application of eflornithine (difluoromethylornithine, DFMO), an inhibitor of ornithine decarboxylase (ODC), the rate limiting enzyme in the polyamine biosynthesis pathway [29], can reduce undesired, excessive hair growth [30], [31]. We have previously shown that addition of DFMO to organ-cultured human scalp HFs shortens the growth phase of the hair cycle (anagen) and inhibits hair shaft production, accompanied by a decrease in matrix keratinocyte proliferation [32]. However, whether and how spermidine itself affects *human* hair growth directly is unknown.

Therefore, we have exploited the HF as an excellent model system for exploring physiological polyamine functions in human skin [29]. Specifically, we have studied in normal, microdissected human scalp HFs under serum-free organ culture conditions [33]–[35] whether spermidine impacts on basic hair biology parameters. We opted for testing the spermidine doses that have been previously shown to modulate wool follicles growth in organ culture [21], and that correspond to the physiological spermidine levels in human plasma [36]. Using keratin 15 (K15) expression and *K15* promoter-driven green fluorescent protein (GFP) expression as a system for assessing human epithelial HF stem cell functions *in situ* and *in vitro* [35], [37], [38], we also explored whether spermidine alters human HF epithelial stem cell clonogenicity, whose modulation by polyamines is as yet unknown [17], [18], [38], [39].

## Results

### Spermidine stimulates hair-shaft elongation and prolongs anagen

**Figure 1:**
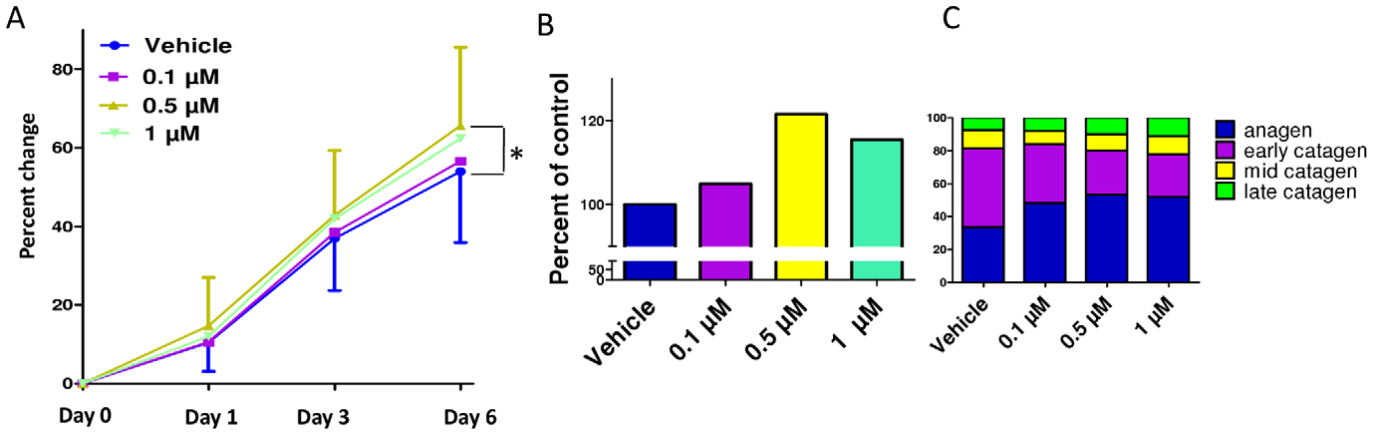
Spermidine treatment increases hair shaft elongation and prolongs anagen. **A.** Spermidine treatment for 6d increased hair shaft elongation of microdissected organ cultured HFs, maximal and significant in the 0.5 µM dose. In this clinically highly relevant *in vitro*-assay, amputated HFs in the growth phase of the hair cycle (anagen) continue for several days to produce a pigmented hair shaft at almost the normal speed of normal anagen HFs *in vivo*, and display cyclic growth activity *in vitro* by spontaneously entering into the regression phase of the hair cycle (catagen). **B.** Total increase in hair shaft elongation relative to control after 6d in culture (cumulative results of two different experiments)**. C.** Spermidine treatment prolonged anagen in all three doses after 6d in culture. *P<0.05. Columns represent means±SEM; n = 30–36 HFs/group; cumulative results of two different experiments.

Administration of spermidine for 6 days slightly, but significantly, increased hair shaft growth of microdissected, organ-cultured normal human scalp HFs (Figure 1A). This effect was maximal and significant at 0.5 µM, and led to more than a 20% increase in hair shaft production after 6 days in culture (Figure 1B).

However, whether spermidine can also prolong the duration of anagen is clinically much more important than the effect on hair elongation since this directly impacts on the amount of hair that is shed (e.g., a reduced percentage of HFs in anagen, clinically, will inevitably result in a higher percentage of catagen and subsequently telogen HFs, thus leading to telogen effluvium – and vice versa) [17], [18], [40]. To assess this, quantitative hair cycle histomorphometry was performed [41], [42]. Indeed, after 6 days of organ culture, all doses of spermidine investigated led to an increase in the percentage of HFs in anagen, and decreased that of catagen HFs (Figure 1C). In the presence of spermidine, only 47–52% of the HFs spontaneously entered catagen, while 67% of the control HFs had already done so.

### Spermidine downregulates ODC expression in the HF

**Figure 2:**
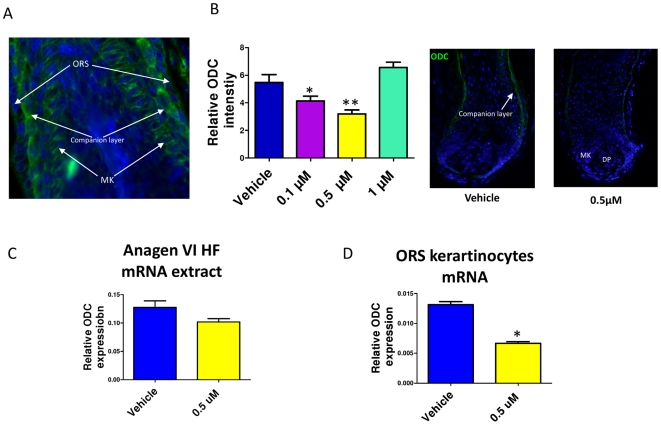
Spermidine treatment down-regulates ODC expression in the HF. **A.** Immunoreactivity of ODC in the proximal HF demonstrates moderate ODC expression in the matrix keratinocytes, and strong expression in the proximal companion layer. **B.** Spermidine treatment for 6d decreased ODC immunoreactivity in the lower doses (0.1 and 0.5 µM). Columns represent means±SEM; n = 9–13 HFs/group **C.** By Q-PCR, spermidine treatment for 24 h slightly decreased the *ODC* transcript steady-state levels in human anagen HF mRNA extracts, compared to *PPIA,* although this did not reach significances. **D.** However, spermidine significantly down regulated *ODC* transcription in cultured human ORS keratinocytes after 48 h. *P<0.05. Columns represent means±SEM. Results represent triplicate determinations of samples. Total RNA was pooled from 20 HFs. DP, dermal papilla; MK, matrix keratinocytes; ORS, outer root sheath.

ODC, the rate-limiting enzyme of polyamine synthesis, reportedly is expressed in highly proliferative hair matrix keratinocytes [29], [43]. We have also demonstrated that ODC is present in the matrix keratinocytes of human anagen VI HFs (Fig. 2A). However, surprisingly, high immunoreactivity was also evident in the companion layer of the HF (Fig. 2A).

Since polyamines negatively regulate ODC activity, both on transcriptional and post-transcriptional level [29], we checked whether any such effect is also apparent in spermidine-treated HFs. We found that spermidine treatment for 6 days downregulated ODC protein expression *in situ* (Figure 2B), and showed a tendency towards decreased *ODC* mRNA transcription after 24 h treatment (Figure 2C), though the latter was not significant. However, in isolated, cultured human HF-derived outer root sheath (ORS) keratinocytes, 0.5 µM spermidine treatment for 48 h significantly downregulated *ODC* mRNA expression (Figure 2D). Together, these data suggest that excess spermidine induces intracellular counter-regulatory events in human HF epithelium *in situ* through which further intrafollicular polyamine synthesis is restricted by inhibiting *ODC* gene and protein expression.

### Spermidine stimulates human hair matrix and epidermal keratinocyte proliferation

**Figure 3:**
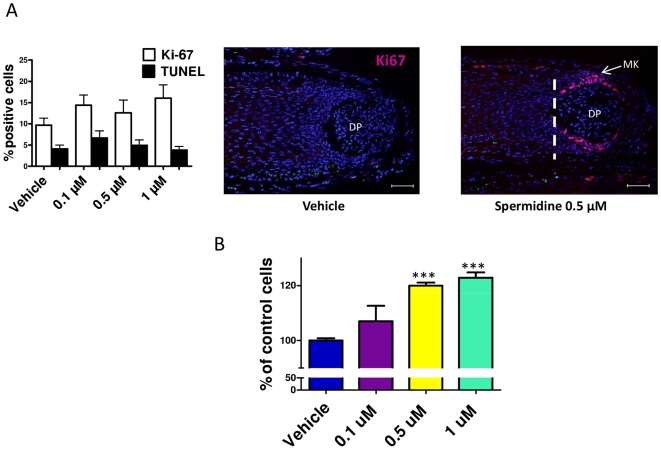
Spermidine stimulated proliferation of primary human keratinocytes and tended to stimulate hair matrix keratinocytes. **A.** By quantitative Ki67/TUNEL immunohistomorphometry, spermidine tended to slightly stimulate hair matrix keratinocyte proliferation *in situ*; however, this did not reach statistical significance. No effect was seen on hair matrix keratinocyte apoptosis *in situ*. Columns represent means±SEM; n = 30–36 HFs/group; cumulative results of two different experiments. **B.** Spermidine significantly stimulated proliferation of primary human keratinocytes after 48 h, as assessed by fluorimetric proliferation assay. The values shown are means±SEM of triplicate experiments. Scale bars, 50 µm. Columns represent means±SEM; ***P<0.001. DP, dermal papilla; MK, matrix keratinocytes.

The fact that spermidine prolonged anagen suggested effects on highly proliferative hair matrix keratinocytes [17], [18]. *In situ*, spermidine slightly, but not significantly stimulated hair matrix keratinocyte proliferation, as assessed by quantitative Ki67-immunohistomorphometry (Figure 3A **)**, and did not significantly alter hair matrix keratinocyte apoptosis (Figure 3A). However, fluorimetric proliferation assays revealed that spermidine dose-dependently stimulated the proliferation of cultured primary human epidermal keratinocytes (Figure 2B). FACS analysis revealed that spermidine promoted the accumulation of cells in the S/G2-M phases of the cell cycle (data not shown). Thus, the promotion of S/G2-M entry by human HF keratinocytes may be one potential mechanism by which spermidine may exert its anagen-prolonging effects.

### Spermidine differentially modulates the gene expression profile of human HFs

In order to identify potential (direct or indirect) target genes of spermidine action in human HFs, a genome-wide microarray analysis was performed on two independent sets of organ-cultured human scalp HFs, derived from two healthy female volunteers (aged 47 and 67 yrs), which had been treated with 0.5 µM spermidine or vehicle for 24 h and 48 h, respectively. In order to check which genes are being constitutively activated following spermidine administration, we made a comparison between these two time-points. Adopting rigid selection criteria, i.e. selecting only those genes whose intrafollicular transcription was modified >2-fold (p < 0.05) into the same direction in both individuals, only five genes were identified as significantly and equidirectionally up-regulated by the tested spermidine dose (Table 1). Of these spermidine-regulated genes, *SYVN1* and *NACA* are known to be important for normal cell homeostasis and function, including endoplasmic reticulum-associated protein degradation [44] and folding and targeting of nascent proteins [45]. *SLC25A3* encodes a mitochondrial phosphate carrier, essential for the aerobic synthesis of adenosine triphosphate [46], and *POP3* is important for cell adherence and migration [47] (see Discussion for details).

**Table 1:** Selected genes up-regulated in HFs by spermidine (0.5 µM).

Gene name	Sequence description	Sample 1		Sample 2	
		Fold change	P-value	Fold change	P-value
*SYVN1*	**Synovial apoptosis inhibitor 1, synoviolin**: Ubiquitin ligase, plays a role in endoplasmic reticulum-associated protein degradation [44].	2.81	0.012	3.74	0.002
*KRT77*	**Keratin 77**: Intermediate filament expressed in the luminal cells of eccrine sweat glands [69]	2.58	0.006	3.24	0.005
*NACA*	**Nascent-polypeptide-associated complex alpha polypeptide**: Part of the protein translation chaperone complex, which has an important role in cotranslational processes, in addition to transcriptional regulation-related processes and cell differentiation [45]	2.1	0.033	2.73	0.007
*POPDC3*	**Popeye domain containing 3**: Takes part in cell adherence and migration, and essential for normal heart function [47], [70]	2.29	0.018	2.09	0.029
*SLC25A3*	**Solute carrier family 25 (mitochondrial carrier; phosphate carrier), member 3**: Mitochondrial phosphate carrier, essential for the aerobic synthesis of adenosine triphosphate [67]	3.74	0.004	2.05	0.026

### Spermidine up-regulates expression of the epithelial stem cell-associated keratins K15 and K19, and modulates K15 promoter activity *in situ*

Anagen maintenance requires constant production of HF keratinocytes from resident epithelial stem cells (eSCs) and their progeny, which are thought to migrate towards the hair matrix [18], [48]. Since polyamines have been shown to be required for the migration [3], [4] and differentiation of various progenitor cell populations [5]–[7], [49] as well as for cell cycle progression [50], [51], we therefore addressed whether spermidine also affects human HF eSCs. These cells are located primarily in the bulge and are characterized by expression of K15; however, K15+ progenitors are also found further down the ORS [17], [18], [38], [39].

**Figure 4:**
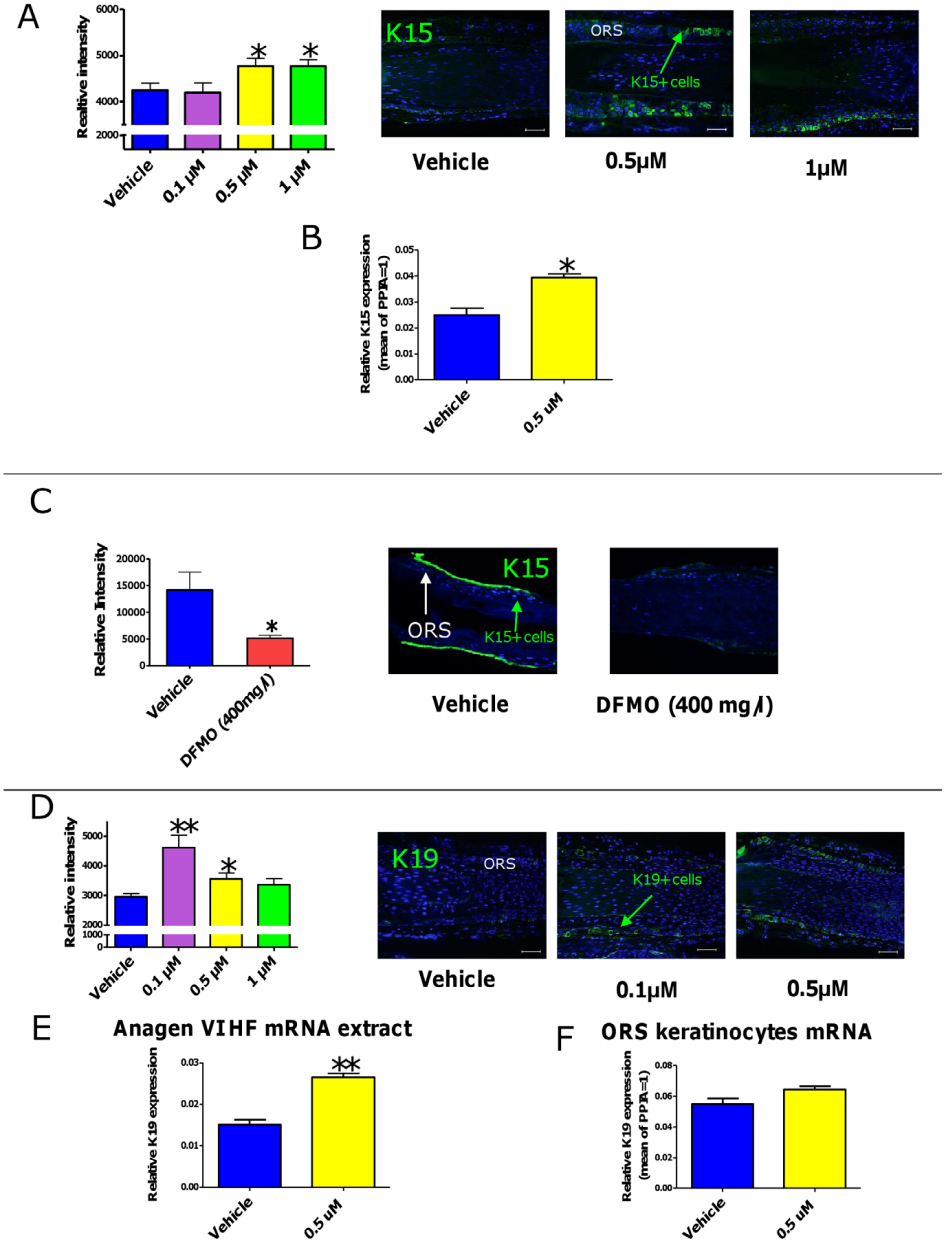
Spermidine up-regulates expression of the epithelial stem cell-associated keratins K15 and K19. **A**. Spermidine treatment for 6d increased K15-like immunoreactivity in the 0.5 µM and 1 µM doses, as assessed by quantitative immunoreactivity. n = 10–15 HFs/group; cumulative results of two different experiments. **B.** Spermidine significantly upregulated *K15* mRNA expression in cultured ORS keratinocytes after 48 h of treatment. Results represent triplicate determinations of samples. Total RNA was pooled from 20 HFs. **C.** DFMO (400 mg/l) significantly decreased K15-like immunoreactivity after 6d. n = 6–8 HFs/group**. D.** Spermidine treatment for 6d increased K19-like immunoreactivity in the 0.1 µM and 0.5 µM doses, as assessed by quantitative immunoreactivity. n =  30–36 HFs/group; cumulative results of two different experiments. **E.** By Q-PCR, spermidine treatment for 48 h significantly increased the *K19* transcript steady-state levels in human anagen HF mRNA extracts. **F.** Treatment of ORS keratinocytes for 48 h showed a tendency of *K19* mRNA upregulation, but these results were not statistically significant. Results represent triplicate determinations of samples. Total RNA was pooled from 20 HFs. Scale bars, 50 µm. Columns represent means±SEM; *P<0.05; **P<0.01. ORS, outer root sheath.

Even though our microarray analysis had not identified any HF-associated keratins as spermidine-regulated genes (possibly for methodological or sub-threshold reasons), quantitative immunohistomorphometry of K15 expression *in situ* clearly showed that spermidine significantly increased K15 immunoreactivity in the basal layer of the proximal ORS after 6 days (Figure 4A).

To confirm this effect in isolated human HF keratinocytes, which are not influenced by the HF mesenchyme, HF melanocytes, and intrafollicular hematopoietic cells, we measured the steady-state levels of *K15* mRNA in cultured human ORS keratinocytes that had been treated for 48 h with 0.5 µM spermidine. Indeed, *K15* mRNA was upregulated after 48 h of treatment (Figure 4B). Conversely, ODC inhibition by DMFO significantly downregulated *K15* expression after 6 days (Figure 4C). This suggests that K15 protein expression *in situ* is profoundly regulated by spermidine.

In the lower two doses tested, spermidine also significantly increased keratin 19 (K19) protein expression after 6 days, an independent human HF epithelial progenitor cell marker [35], [52] (Figure 4D). Additionally, 0.5 µM spermidine administration led to increased *K19* mRNA expression in organ-cultured HFs after 24 h (Figure 4E). Treatment of cultured ORS keratinocytes with 0.5 µM spermidine tended to upregulate *K19* mRNA after 48 h, but this was not statistically significant (Figure 4F).

**Figure 5:**
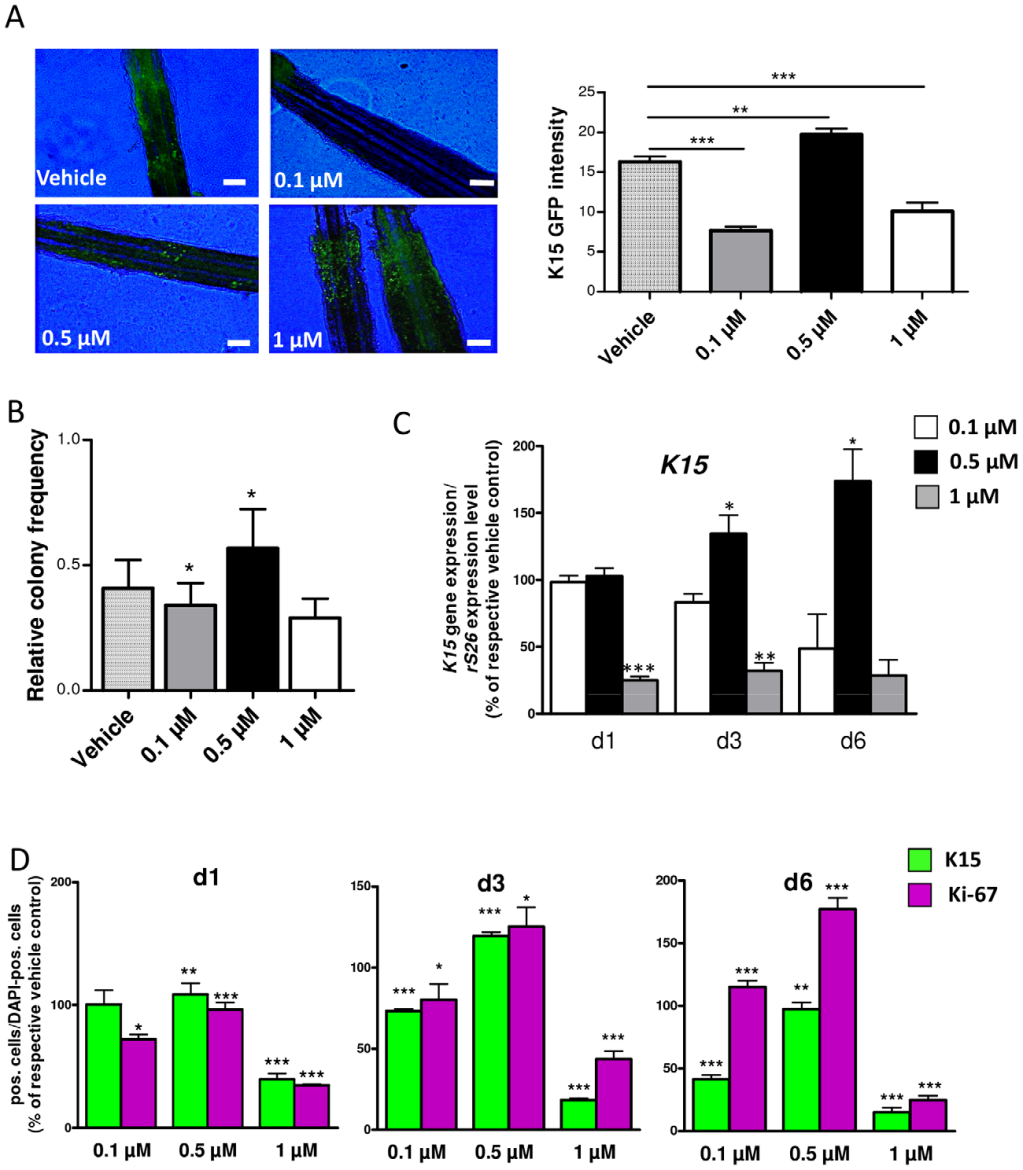
Spermidine modulates colony forming efficiency and K15 expression of isolated K15-GFP+ cells *in vitro*. **A.** Spermidine regulates expression of *K15* promoter-driven GFP expression in hair follicles *in situ* in a dose-dependent manner; scale bars, 100 µm. **B.** Colony formation assay of isolated and cultured K15-GFP+ under different spermidine concentrations revealed that 0.5 µM spermidine significantly upregulated colony forming efficiency, whereas 0.1 and 1 µM reduced it. **C.** Quantitative RT-PCR of *K15* in isolated and cultured K15-GFP+ cells under the presence of spermidine**.** *K15* expression is presented in comparison to the housekeeping gene *rS26*. **D.** Evaluation of proliferative and apoptotic events in isolated and cultured K15-GFP+ cells under the influence of spermidine for 6 days. At three time points (d1, d3, and d6) cells were labeled with K15, Ki-67 and DAPI. K15- and Ki-67-positive cells were counted and expressed as percentage of all DAPI-positive cells (nuclei). Error bars represent means±SEM; data were analysed by unpaired Student's t-test (vehicle group vs. test group; A, C) and two-way ANOVA test (within corresponding groups; B, D);*P<0.05; **P<0.01;***P<0.001.

To further explore the effect of spermidine on human HF eSCs in the bulge [39], [48], we employed a novel human *K15* promoter-driven GFP reporter assay, which demarcates K15+ human HF epithelial progenitor cells *in situ* [37], [38]. This revealed that spermidine also significantly and dose-dependently modulated human *K15* promoter activity in the bulge region *in situ*: As measured by the intensity of GFP fluorescence, at 0.5 µM spermidine up-regulated the transfected *K15* promoter activity *in situ*, while it down-regulated it at 0.1 µM or 1 µM (Figure 5A).

### Spermidine dose-dependently modulates colony forming efficiency and K15 expression of isolated K15-GFP+ cells *in vitro*

To further investigate how spermidine impacts on human HF epithelial progenitor cells in the absence of HF mesenchyme, HF melanocytes and intrafollicular hematopoietic cells, we isolated and cultured human HF K15-GFP+ progenitor cells [37], [38]. These experiments showed that only the 0.5 µM spermidine dose significantly up-regulated the colony forming efficiency of K15-GFP+ progenitor cells (Figure 5B). This spermidine dose also increased their *K15* mRNA expression on days 3 and 6 (Figure 5C), and slightly enhanced their K15 protein expression on days 1 and 3 (Figure 5D). Furthermore, 0.5 µM spermidine also stimulated proliferation on days 3 and 6 of these isolated human HF progenitor cells *in vitro* (Figure 5D). In contrast, 1 µM spermidine significantly inhibited *K15* mRNA expression and the proliferation activity of K15-GFP+ cells *in vitro* (Figures 5C, D).

These *in vitro* results are comparable with the *in situ* observations, which also showed maximal spermidine growth-modulatory effects on the intact HF mini-organ at the same dose. Taken together, this suggests that the spermidine-induced growth-promotion of human HFs at 0.5 µM (Figures 1A, B) may be mediated, at least in part, by an effect on epithelial HF progenitor cells, which are also stimulated by the same dose.

## Discussion

While it has long been postulated that polyamines may stimulate hair growth [22], [29], [53], the current study is the first to provide direct evidence that this is actually the case in human HFs. Specifically, we demonstrate that spermidine stimulates hair shaft elongation, accompanied by prolongation of anagen, and thus directly promotes human HF growth. This is in line with recent *in vivo* evidence that topically applied alpha-methylspermidine induces anagen in mouse telogen HFs [22].

The anagen prolongation/catagen inhibition by spermidine demonstrated here is clinically important: If our human HF organ culture data are transferrable to the *in vivo* situation, spermidine administration may help to counteract multiple forms of hair loss associated with excessive hair shedding. Namely, topical or nutraceutical spermidine application may reduce telogen effluvium in patients that suffer from hair loss due to premature anagen termination (e.g. by androgens, perifollicular inflammation, iron or estrogen deficiency, or effluvium-inducing drugs) [17], [54].

ODC is expressed in the anagen hair matrix [29], [55], and reducing intrafollicular spermidine synthesis by the inhibition of ODC activity reduces human hair shaft growth and shortens anagen duration [32]. Together with the anagen-prolonging and hair growth-promoting effects of spermidine demonstrated here, this suggests that intrafollicular synthesis of spermidine is important for keeping a HF in its growth stage. However, over-expression of ODC in mice results in the formation of dermal cysts, leading to the opposite effect ( = hair loss) [20], [25]. This is postulated to result from the excessive accumulation of putrescine, which causes disturbed keratinocyte differentiation and increased proliferation. In fact, we show here that spermidine application downregulates intrafollicular ODC expression on the gene and protein levels (Fig. 2B-D), and may thus protect the human scalp HF against excessive synthesis of putrescine so as to avoid deleterious polyamine effects on human hair growth.

To our surprise, although also evident in the proliferating keratinocytes of the hair bulb, the strongest expression of ODC was evident in the companion layer of the HF (Fig. 2A). This expression pattern coincides with the intriguing pattern of expression of K6/K16, the keratins known to be expressed in highly proliferating cells [56]. Thus, our findings add to the mystery which still surrounds this layer in the HF, and which awaits further investigation.

Despite the known general proliferation-stimulatory properties of polyamines [57], [58], which were confirmed here in cultured human epidermal keratinocytes (Fig. 3B), and the demonstrated inhibitory effects of DFMO on human hair matrix keratinocytes [32], spermidine exerted only minor effects on hair matrix proliferation *in situ*. Therefore, much of the stimulatory effect of spermidine on hair shaft production appears to arise from a prolongation of anagen (Fig. 1C). This supports the concept that polyamines are potent regulators of HF *cycling*, not only in mice [19], [22], [26], but also in man. It also provides the first evidence that one defined polyamine, spermidine, alters HF cycling by *direct* effects on the HF which are independent of *extrafollicular* changes induced in the concentration of various polyamines and/or in the activity of their multiple different target genes.

The recent provocative report that spermidine may promote longevity [13], [59] may be relevant in the current context, if one considers that, with the notable exception of its pigmentary unit, the HF is one of the most strikingly aging-resistant organs of the human body [60]. Furthermore, the duration and prolongation of anagen is a very faithful indicator of HF vitality [32], [61]. Therefore, one wonders whether spermidine impacts positively on a HF's individual lifespan. Since the latter vitally depends on its epithelial stem cell compartment [48], [62], this raises the question whether the normal function of HF epithelial stem cells depends on proper intrafollicular spermidine synthesis and/or availability.

Though our pilot study was not designed to provide definitive proof for the validity of this hypothesis, our current data suggest that human HF epithelial stem cells are profoundly modulated by spermidine: Spermidine not only upregulates K15 and K19 protein expression *in situ* (Fig. 4A,D), but intrafollicular spermidine synthesis is also needed for normal K15 expression (Figure 4C). Moreover, one tested spermidine dose (0.5 µM) stimulated *K15* promoter activity and colony forming efficiency and long-term proliferation of isolated, primary human HF epithelial progenitors (Fig. 5A,B,D). Our observation that the highest tested dose of spermidine (1 µM) inhibited colony forming efficiency, *K15* promoter activity, and proliferation at day 6, might well result from the metabolism of excess spermidine to toxic compounds that enhance oxidative damage, such as hydrogen peroxide [29].

Although strong upregulation of K15 was evident in all of our experiments at the 0.5 µM dose, K15 protein expression of K15-GFP+ cells *in vitro* was surprisingly downregulated on day 6 (Fig. 5D). This was in contrast to the upregulation of the corresponding mRNA expression that was observed at the same dose (Fig. 5C). It is possible that the rapid proliferation of the K15-GFP+ cells observed after 6 days of 0.5 µM spermidine administration (as assessed by Ki-67 immunoreactivity, Fig. 5D) did not allow enough time for the K15 protein to assemble in the cells, and to reach the level of detection. It has also been shown before that polyamines have an effect on the post-translational regulation of proteins, affecting protein degradation by direct or indirect effect on proteases [63]. Therefore, changes in protein expression may not always correlate with the changes observed at the mRNA level.

Our study presents the first evidence that spermidine is a novel determinant in human eSCs biology, most notably of K15 and K19 expression by primary human epithelial progenitor cells *in situ* and *in vitro*. These findings are in line with the prior demonstration that ODC is expressed in the bulge region of the HF [55], [64], where it colocalizes with that of K15 and K19 expression [52]. While polyamines are known to affect the keratin composition of wool follicles [21], it was previously unknown that polyamines actually regulates the expression of human eSC-associated keratins.

Moreover, we provide the first available evidence that inhibiting the key enzyme of polyamine synthesis (ODC) down-regulates K15 expression. Thus K15 expression *in situ* is profoundly regulated by spermidine, and both polyamines and ODC activity impact on the expression of this HF epithelial progenitor cell marker keratin. Our finding that ODC expression on the gene and protein level (and thus likely ODC activity) underlies a negative, dose-dependent feedback regulation by spermidine (Fig. 2B) underscores the apparent importance of keeping intrafollicular polyamine synthesis in check. That the highest dose of spermidine tested did not reduce ODC expression (Figure 1B) may suggest that adequate ODC activity remains needed as a part of the biological stress response to excessive spermidine levels.

We had hoped to obtain specific leads from our microarray analysis on how spermidine may exert its anagen-prolonging, stem cell-modulatory, and K15/K19-regulatory effects. While these results identified five novel intrafollicular *candidate* target genes for spermidine-mediated signaling (Table 1) that have not yet been investigated in the spermidine literature, these genes do not sufficiently explain the underlying mechanisms of action. However, the fact that the identified candidate genes are important for vital cell organelles and cell homeostasis fits well to the general concept that spermidine supports HF and eSC vitality.

For example, synoviolin (coded by *SYVN1*) is a ubiquitin ligase, which plays an important role in endoplasmic reticulum-associated protein degradation [44], the *NACA* gene encodes the nascent-polypeptide-associated complex alpha polypeptide, a part of the protein translation chaperone complex [45], and *SLC25A3* is a mitochondrial phosphate carrier, which is essential for the aerobic synthesis of adenosine triphosphate [65]. Particularly interesting potential spermidine target gene is *POP3*, which belongs to the highly conserved popeye domain-containing family, and has been implicated in cell adherence and migration [47]. Although these microarray results could not be further validated since all available HF samples and sections had been consumed for the analyses reported here, our preliminary data provide new leads to previously unsuspected (direct or indirect) spermidine target genes in human tissue physiology.

An intriguing chance observation of our study was the finding that spermidine clearly up-regulated transcription of K77 in the HFs of two female patients (Table 1). Since this keratin has previously been claimed to be exclusively expressed in eccrine glands, we are now following this lead up on the gene and protein level in order to obtain deeper insights into the unexpected and enigmatic functions that K77 may have in human HF biology, and why expression of this gene is so spermidine-sensitive.

In summary, our study provides the first evidence that spermidine directly impacts on the growth, cycling, keratin expression and epithelial progenitor functions of human HFs. Due to its anagen-prolonging effects, spermidine deserves rigorous clinical testing as a candidate anti-hair loss agent. It could become an adjuvant therapy for hair loss disorders associated with premature catagen induction, leading to telogen effluvium, and/or reduced hair shaft production. Moreover, we show that the complex regulatory role of polyamines in human epithelial biology *in situ* extends far beyond the mere stimulation of proliferation. Our study also documents that, to further dissect the full range of polyamine functions in normal human tissue physiology, human HF organ culture offers a highly instructive, clinically relevant research tool (34).

## Materials and Methods

### HF organ culture

Anagen VI HFs were microdissected from normal temporofrontal human scalp skin obtained after written informed patient consent from ten healthy adult females undergoing routine facelift surgery, adhering to the Helsinki guidelines and following approval by the Institutional Research Ethics Committee of the University of Lübeck. In this clinically highly relevant *in vitro*-assay, amputated HFs in the growth phase of the hair cycle (anagen) continue for several days to produce a pigmented hair shaft at almost the normal speed of normal anagen HFs *in vivo*, and display cyclic growth activity *in vitro* by spontaneously entering into the regression phase of the hair cycle (catagen). For immunohistochemical studies, isolated HFs from four individuals were used for routine 6-day HF organ culture as previously described [17], [18], [32]-[35]. Spermidine (0.1, 0.5 or 1 µM), DFMO (400 mg/l) or vehicle (distilled water) were administered once for each change of culture medium (every 48 h). After 6 days, all test and control HFs were embedded, snap-frozen, and processed for longitudinal cryosectioning. The frozen sections were stored at −80°C until used. HFs from one individual were used for a 24 h organ culture, and utilized for a genome-wide microarray essay and quantitative PCR (qPCR). HFs from four additional individuals were subjected to 48 h organ cultures. HFs from two individuals were used for *K15* promoter-driven green fluorescent protein (GFP) expression construct transfection (see below). HFs from an additional one female patient were used for a genome-wide microarray essay. HFs from one individual were used for isolating and culturing of K15-GFP positive cells.

### Measurement of hair-growth parameters

Hair-shaft length measurements of vehicle and spermidine-treated HFs in organ culture was performed on d 0, 1, 4 and 6, using a Zeiss inverted binocular microscope with an eyepiece containing a graticule (Carl Zeiss, Oberkochen, Germany). It should be noted that hair-shaft elongation in serum-free human HF organ culture occurs at almost the same rate of normal hair growth *in vivo* [34]. HF cycle staging (*i.e.*, anagen VI *vs.* catagen) was assessed histologically after 6 days of organ culture using hematoxylin–eosin (Sigma-Aldrich, Taufkirchen, Germany) stained frozen sections. The hair cycle stage was classified according to accepted morphological criteria [32].

### Analysis of HF matrix keratinocyte proliferation and apoptosis

To evaluate apoptotic cells in colocalization with a proliferation marker Ki-67 of cells in the HF, Ki-67/TUNEL (terminal dUTP nick endlabeling) double-immunofluorescence was used as described previously [33]. Briefly, 6- µm cryosections were fixed in paraformaldehyde and ethanol-acetic acid (2∶1) and labeled with a digoxigenin-deoxyUTP (ApopTag Fluorescein In Situ Apoptosis Detection Kit; Chemicon, Purchase, NY, USA) in the presence of terminal deoxynucleotidyl transferase (TdT), followed by incubation with a mouse anti-Ki-67 antiserum (Dako, Glostrup, Denmark). TUNEL-positive cells were visualized by an anti-digoxigenin FITC-conjugated antibody (ApopTag kit), whereas Ki-67 was detected by a rhodaminelabeled goat anti-mouse secondary antibody (Jackson Immuno- Research, West Grove, PA, USA). Counterstaining was performed with 4′,6-diamidino-2-phenylindole (DAPI) (Roche Molecular Biochemicals GmbH, Mannheim, Germany). Negative controls were run by omitting TdT or the primary Ki-67 antibody and by observing negative immunoreactivity for these parameters in the expected human scalp skin compartments. For the quantitative evaluation and comparison of the double-staining, DAPI-, Ki-67-, or TUNEL-positive cells were counted in clearly defined reference regions. The number of DAPI-positive cells served as “total number of cells,” and the percentage of Ki-67-positive and/or TUNEL-positive cells was calculated on this basis to enable comparison between vehicle and test groups.

### ORS keratinocyte culture

Plucked HFs were digested using trypsin to obtain human ORS keratinocytes, as described previously [66]. ORS cultures were kept on a feeder layer of mitomycin-treated human dermal fibroblasts [67] in a 1∶3 mixture of Ham's F12 (Biochrom, Berlin, Germany) and Dulbecco's modified Eagle's medium (Invitrogen-Life Technologies, Inc., Grand Island, NY, USA) supplemented with 0.1 nM cholera toxin, 5 µg/ml insulin, 0.4 µg/ml hydrocortisone, 2.43 µg/ml adenine, 2 nM triiodothyronine, 10 ng/ml epidermal growth factor, 1 mM ascorbyl-2-phosphate, and antibiotics (all from Sigma-Aldrich Corp.).

### Quantitative immunohistochemistry of K15, K19 and ODC

Expression of K15 and K19 was studied using our previously described tyramide signal amplification protocol and basic immunohistology protocols [35]. For ODC immunohistochemistry, rabbit anti-human ODC antibody (Abnova, 1∶500) was used. As negative controls for all stainings, the respective primary antibodies were omitted. In addition, morphological criteria and reproduction of the previously published HF expression pattern of K15 and K19 was used as internal positive or negative controls [35], and for ODC, expression pattern in the epidermis was used as a positive control. Densitometric measurements of staining intensities in previously defined reference areas (quantitative immunohistomorphometry) we performed using the ImageJ software (National Institute of Health, Bethesda, USA), as previously described [33]. Statistical analysis was assessed by unpaired *t* test, using Graph Pad Prism 5.01 (Graph Pad Software, Inc., San Diego, CA, USA).

### Microarray

Gene expression analysis of HFs from two female subjects (50 HFs from each subject) using an Agilent Human Whole Genome Oligo Microarray (44K) was performed by Abiol Ltd. (Debrecen, Hungary) as a commercial service (. Freshly isolated HFs (25/group, all derived from a single donor) were treated with vehicle or 0.5 µM spermidine, and total RNA was isolated according to standard protocols (TRIzol; Invitrogen-Life Technologies). The quality of total RNA was controlled via an Agilent 2100 Bioanalyzer System (Agilent Technologies, Santa Clara, CA, USA). Linear amplification of RNA and hybridization of the whole genome oligo microarray were performed according to the manufacturer's protocols (Agilent Technologies). For analysis, after rejection of outlier features, the expressed genes showing at least 2.0-fold differential expression were further analyzed by a statistical test using the Cross-Gene Error Model based on Deviation from one (recommended by Agilent Technologies). Candidate genes were selected according to the following criteria: equidirectional expression changes in both examined individuals, value of *P* < 0.05, and changes ≥2-fold. All data presented in this study is MIAME compliant and the raw data have been deposited in a MIAME compliant database, the NCBI's Gene Expression Omnibus (GEO).

### qPCR

Analysis of expression of specific mRNA transcripts of keratins *K15*, *K19* and of *ODC* was performed by quantitative real-time PCR using the 5′ nuclease assay (ABI PRISM 7000 Sequence Detection System, Applied Biosystems, Foster City, CA, USA). Total RNA was isolated from whole HFs using TRIzol and digested with RQ1 RNase-free DNase (Promega, Madison, WI, USA) according to the manufacturer's protocol. After isolation, 1 µg of RNA was reversetranscribed into cDNA using 15 U of avian myeloblastosis virus reverse transcriptase (Promega) and 0.025 µg/µl random primers (Promega). PCR amplification was performed using TaqMan primers and probes (assay identification number: Hs00267035_m1 for *K15*, Hs01051611_gH for *K19*, and Hs00189739_m1 for *ODC*) using the TaqMan Gene Expression Master Mix Protocol (Applied Biosystems). A transcript of *cyclophilin A* (*PPIA*, Hs99999904_m1) was used as a housekeeping gene control. The amounts of the above-mentioned transcripts were normalized to those of the control genes using the ΔΔCt method. During normalization, the geometric mean of the Ct values of the four endogenous controls (provided that the SD of Ct values of the individual housekeeping genes, obtained in triplicate determinations, was <0.5) was used to normalize gene expression of the given gene of interest.

### Isolation and in vitro studies with K15-GFP+ cells

#### Transfection of isolated hair follicles

Scalp skin was dissected into approximately 0.5 cm^2^ pieces, which were washed with PBS (37°C) to remove cell debris. These pieces were incubated overnight in Williams E Medium (Biochrom, Cambridge, UK) supplemented with 0.01% w/v reconstituted Dispase® (Invitrogen-Gibco, Grand Island, NY, USA) at 4°C. After washing with PBS, the epithelial core of single, full-length HFs were carefully plucked out from the tissue fragments with microdissection tweezers, leaving the HF dermal sheath retained in the skin. For further experiments, the HFs were collected and cultured in K-SF medium (Invitrogen-Gibco, Carlsbad, CA, USA) or directly transfected with a human *K15* promoter-driven GFP expression construct as described [37], [38]. Direct immunofluorescence microscopic analysis was used to evaluate the expression of GFP. As negative controls, Dispase® treated, microdissected and only Lipofectamine® (Invitrogen, Karlsruhe, Germany) treated HFs were used. For positive controls an ubiquitously GFP expressing construct was used to transfect the HFs. All samples were viewed and photographed using a Keyence BZ 8000 fluorescence microscope. Densitometric measurements of GFP intensity and statistical analysis were performed as previously described [37], [38].

#### Isolation, selection and culture of human K15-GFP+ cells

To isolate and culture K15-GFP+ cells positively transfected HFs were processed as described previously [37], [38]. To test the influence of spermidine, it was added to the K15-GFP+ cell culture with an adjusted concentration of 0.1, 0.5, and 1 µM.

#### Semi- and quantitative RT-PCR for expression analysis of K15

Total RNA was isolated from an equivalent number of control and K15-GFP+ cells (∼10.000 cells/dish) of vehicle and spermidine treated K15-GFP+ cells by using RNeasy Mini Kit (Qiagen, Germany) according to the manufacturer's protocol. Total RNA (100 ng) was reversed transcribed in 20 µl using the Perkin-Elmer RNA PCR kit (Branchburg, NJ). For quantitative analysis of *K15* and the housekeeping gene *ribosomal protein S26 (rS26),* parts of the cDNA products (2 µl) were subjected to PCR analysis. *K15* cDNA was amplified with primers *K15*-f (5′-GGAGGTGGAAGCCGAAGTAT-3′) and *K15*-r (5′-GAGAGGAGACCACCATCGCC-3′). For comparative analysis *rS26* was amplified with primers *rS26*-f (5′-CCGTGCCTCCAAGATGACAAAG-3′) and *rS26*-r (5′-ACTCAGCTCCTTACATGGGCTT-3′). For quantitative qPCR expression analysis of *K15*, *GFP* and *rS26* 100 ng RNA was reverse transcribed in a total of 20 µl using the LightCycler® RNA-Master-SYBR-Green-I-kit and the LightCycler® 2.0 instrument (Roche). For qPCR analysis of *K15* and *rS26* cDNA was amplified with primers as described above. Vehicle treated K15-GFP+ cells were used as controls. For quantification analysis the LightCycler® Software 4.05 (Roche) was used. The results represent three independent experiments and data expressed as means±SEM.

#### Colony forming efficiency

For colony forming efficiency assays under the influence of spermidine K15-GFP+ cells were seeded onto a 35 mm culture dish coated with collagen IV and fibronectin (100 cells/well). K15-GFP+ cells were cultured as previously described [37], [38]. The analysis of the clonal potential was carried out as previously described [37], [38]. Vehicle treated K15-GFP+ cells were used as controls. The results represent 3 independent experiments and data expressed as means±SEM.

#### Cell proliferation

For the detection of proliferating K15-GFP+ cells after 1 day, 3 days, and 6 days under spermidine treatment, K15-, Ki-67-, and DAPI labeling was performed as described previously [37], [38]. Vehicle treated K15-GFP+ cells were used as control for the Ki-67 reaction. The results were expressed as mean percentage of treated cells compared to the vehicle control sample of 3 independent experiments and data expressed as means±SEM.

### Cell proliferation assays

Primary human keratinocytes were isolated as described by Rheinwald and Green [68] from fronto-temporal scalp skin specimens, obtained with written informed consent during routine face-lift surgery (Caucasian females over 45 y). The East London and City Health Authority Research Ethics Committee approved the use of redundant human skin for this study. After isolation, keratinocytes were cultured in keratinocyte serum free medium (K-SFM) (Invitrogen, Paisley, UK) at 37°C in a humidified atmosphere of 5%CO_2_/95% air. Spermidine (Sigma-Aldrich, Poole, UK) was added to the culture medium at 0.1, 0.5, and 1.0 µM concentrations for 48 h. After 48 h cell proliferation was assayed using fluorometric reagent Cell Quanti Blue (BioAssay Systems, CA USA). The CellQuanti Blue reagent was diluted 1 in 10 in K-SFM and added to the cells for 1 h. Fluorescence was measured using a Bio Tek Synergy HT plate reader with KC4 software (Bio Tek, VT, USA) with a 530 nm excitation filter and a 590 nm emission filter.

### Cell cycle analysis by propidium iodide flow cytometry

After 24 h +/− spermidine primary human keratinocytes were trypsinised, washed with PBS and fixed in 70% ethanol at 4°C overnight. Cells were resuspended in 50 µg/ml propidium iodide(PI) (Sigma-Aldrich), 100 µg RNaseA (Sigma-Aldrich), 3.8 mM sodium citrate in PBS and incubated at 4°C over night. For each sample 100000 cells were analysed using a LSRII flow cytometer with FACSDiva software (v 5.1.03) (both BD Biosciences, CA USA). The detector was set to linear, PI excitation at 488 nm and emission collected in the 610–620 channel, cells were gated on width and area to exclude doublets.
